# Impact of Donor Milk on Short- and Long-Term Growth of Very Low Birth Weight Infants

**DOI:** 10.3390/nu11020241

**Published:** 2019-01-22

**Authors:** Rebecca Hoban, Michael E. Schoeny, Anita Esquerra-Zwiers, Tanyaporn K. Kaenkumchorn, Gina Casini, Grace Tobin, Alan H. Siegel, Kousiki Patra, Matthew Hamilton, Jennifer Wicks, Paula Meier, Aloka L. Patel

**Affiliations:** 1 Department of Paediatrics, Division of Neonatology, Hospital for Sick Children, Toronto, ON M5G 1X8, Canada; 2 Department of Pediatrics, Section of Neonatology, Rush University Medical Center, Chicago, IL 60612, USA; kousiki_patra@rush.edu (K.P.); paula_meier@rush.edu (P.M.); aloka_patel@rush.edu (A.L.P.); 3 College of Nursing, Rush University Medical Center, Chicago, IL 60612, USA; Michael_schoeny@rush.edu; 4 Nursing Department, Hope College, Holland, MI 49423, USA; aezwiers@hope.edu; 5 Division of Gastroenterology, Seattle Children’s Hospital, Seattle, WA 98105, USA; tanyaporn.kaenkumchorn@seattlechildrens.org; 6 Rush Medical College, Rush University, Chicago, IL 60612, USA; gina_t_casini@rush.edu (G.C.); grace_e_tobin@rush.edu (G.T.); alan_h_siegel@rush.edu (A.H.S.); 7 Department of Pediatrics, Division of Neonatology, The University of Texas Health Science Center at Houston, Houston, TX 77030, USA; Matthew.C.Hamilton@uth.tmc.edu; 8 Department of Pediatrics, Division of Hospital-Based Medicine, Ann & Robert H. Lurie Children’s Hospital of Chicago, Chicago, IL 60611, USA; jennifer.wicks@northwestern.edu

**Keywords:** human milk, breastfeeding, neonatal, donor milk, growth, very low birth weight

## Abstract

Mother’s own milk (MOM) reduces the risk of morbidities in very low birth weight (VLBW) infants. When MOM is unavailable, donor breastmilk (DM) is used, with unclear impact on short- and long-term growth. This retrospective analysis compared anthropometric data at six time points from birth to 20–24 months corrected age in VLBW infants who received MOM supplements of preterm formula (*n* = 160) versus fortified DM (*n* = 161) during neonatal intensive care unit (NICU) hospitalization. The cohort was 46% female; mean birth weight and gestational age (GA) were 998 g and 27.3 weeks. Multilevel linear growth models assessed changes in growth *z*-scores short-term (to NICU discharge) and long-term (post-discharge), controlling for amount of DM or formula received in first 28 days of life, NICU length of stay (LOS), birth GA, and sex. *Z*-scores for weight and length decreased during hospitalization but increased for all parameters including head circumference post-discharge. Short-term growth was positively associated with LOS and birth GA. A higher preterm formula proportion, but not DM proportion, was associated with slower rates of decline in short-term growth trajectories, but feeding type was unrelated to long-term growth. In conclusion, controlling for total human milk fed, DM did not affect short- or long-term growth.

## 1. Introduction

Mother’s own milk (MOM) is the recommended form of nutrition for all infants, particularly for those born preterm, in whom MOM reduces the risk of neonatal morbidities, including necrotizing enterocolitis (NEC), and improves long-term neurodevelopmental outcomes [1,2,3]. With increased awareness of these outcomes, when MOM is of insufficient quantity to support the preterm infant, pasteurized banked breast milk (donor milk (DM)) is often used in neonatal intensive care units (NICUs) as a second-best alternative to avoid the use of formula [4,5,6]. DM, like MOM, has been shown to lower the risk of NEC compared to formula but does not reduce the risk of the many other morbidities for which MOM is protective [3,7,8,9].

DM has been associated with decreased rates of short-term, in-hospital growth compared to both preterm formula and MOM [4,7,10,11,12,13]. However, the results have been inconsistent, with a recent randomized trial of fortified DM demonstrating no negative impact on short-term growth [9] and a retrospective study demonstrating better short-term weight gain and head circumference (HC) growth with MOM supplemented with fortified DM rather than MOM plus formula [14]. A limitation in many of these studies is that the proportions of MOM, DM, and formula received by the infant have not been reported. Despite the increasing use of DM in NICUs globally, there is a paucity of studies addressing long-term growth in DM-fed premature infants [7,8,12,15]. Therefore, we sought to evaluate the relationship between type of in-NICU enteral feeding (proportions of MOM, DM, or preterm formula), and weight, length, and head circumference (HC) anthropometric *z*-scores in very low birth weight (VLBW, birth weight <1500 g) infants at six time points: Birth, 32 weeks corrected gestational age (CGA), NICU discharge, and 4, 8, and 20–24 months corrected age (CA) at NICU clinic follow-up visits. We hypothesized that DM-fed infants would have persistently decreased anthropometric *z*-scores post-discharge in comparison to infants supplemented with formula.

## 2. Materials and Methods

### 2.1. Sample

The sample included 321 VLBW infants admitted to an urban level III NICU in Chicago in two time periods: A “pre-DM” group using preterm infant formula as a supplement to MOM if MOM was unavailable/insufficient in quantity, and a “DM” group that supplemented MOM with pasteurized DM instead of formula. Data from two cohorts of infants were combined for this comparison: 160 infants admitted between January 2011 and December 2012 who were part of a U.S. National Institute of Health-funded prospective cohort study, for whom inclusion and exclusion criteria have been detailed previously [16,17], comprised the pre-DM group, and 161 VLBW infants admitted between April 2013 and December 2014, for whom data were collected retrospectively, comprised the DM group. Included infants were <32 weeks gestational age (GA) and <1500 g at birth and received post-discharge growth assessments at 4, 8, and 20–24 months CA in the NICU’s high-risk follow-up clinic. The prospective study was approved by the institutional review board (IRB), in which written consent was obtained for mothers and their infants. The IRB approved the retrospective study, including its consent exemption.

### 2.2. Design

This secondary analysis of cohort data compared growth outcomes for the pre-DM and DM groups. In both groups, parenteral nutrition began on the day of birth, with unchanged composition and protocols between the pre- and DM groups. Parental nutrition was weaned as feedings were advanced until discontinuation when feedings reached 120mL/kg/day. In the pre-DM group, enteral feedings were started as soon as MOM was available, a practice that may have delayed feeding initiation for up to 5 days post-birth. If MOM was unavailable by that time, 20 kcal/oz (0.67 kcal/mL) preterm formula was begun. In the DM group, enteral feedings were also initiated as soon as MOM was available, but by day 2 post-birth, DM consent was sought if MOM was unavailable in sufficient quantities. Pasteurized DM from a milk bank in Indiana (The Milk Bank, Indianapolis, IN, USA) was used to supplement insufficient MOM through 35 weeks corrected GA (CGA), following a gradual wean to preterm formula starting at 34 weeks CGA. Preterm formula was used if families did not provide DM consent. In both groups, formula was changed from 20 kcal/oz (0.67 kcal/mL) to standard 24 kcal/oz (0.8 kcal/mL) preterm formula once feedings reached 140 mL/kg/day and caloric density was further increased if needed to maintain adequate growth, at the clinical team’s discretion. Bovine-based human milk (HM) fortifier (Similac HM fortifier, Abbott Laboratories, Abbott Park, IL, USA), was added to both MOM and DM when feeding volumes reached 140 mL/kg/day to approximate 24 kcal/oz (0.8 kcal/mL), assuming a baseline of 20 kcal/oz (0.67 kcal/mL). Macronutrient analysis of MOM or DM was not performed. Additional modular protein (0.5–1 g/kg/day) was routinely provided to all infants receiving DM but was added to MOM only if needed for poor growth and/or low blood urea nitrogen (<9 mg/dL) [18]. Standard full feeding volume was 160 mL/kg/day unless modified by the clinical team. Infants were discharged on unfortified MOM and/or transitional 22 kcal/oz (0.73 kcal/mL) formula and did not have specific nutritional management or nutritionist follow-up after NICU discharge.

### 2.3. Measures

Data were abstracted from the primary study and electronic medical records (EMR). Infant demographics (length of stay (LOS), GA at birth, birth weight, sex, race/ethnicity) and daily enteral intake (volume in mLs of MOM, DM, and/or preterm formula) were collected for the first 28 days of life (DOL) during the NICU hospitalization. The proportion of each type of enteral intake was calculated for each infant as the cumulative volume of MOM, DM, or preterm formula received divided by total volume of enteral feeds (MOM + DM + formula) received over the study time period. Weight (g), length (cm), and HC (cm) were recorded at birth, at 32 weeks CGA, at NICU discharge, and at NICU clinic follow-up visits at 4, 8, and 20–24 months CA. Parent-reported post-discharge liquid diet (MOM, formula, and/or cow’s milk) was collected from the EMR from NICU follow-up clinic visits.

### 2.4. Data Analysis

Infants’ weight, length, and HC measures were converted to *z*-scores using Olsen growth charts for premature infants through 40 weeks CGA and World Health Organization (WHO) growth charts thereafter [19,20]. Corrected age (chronological age in months—((40-GA in weeks)/4)) was used for clinic visit *z*-scores. Small for gestational age (SGA) was defined as birth weight below the 10th percentile [19]. All variables were checked for normality with Shapiro–Wilk. Proportions of MOM, DM, and formula were highly skewed and could not be transformed to achieve normality. Among the other variables, only LOS was determined to be moderately skewed. Although square-root transformation achieved normality, the results did not change using the transformed versus original scaling; therefore, the untransformed version was included in all analyses for ease of interpretation. Quantities and proportions of MOM, DM, and formula and morbidities were compared for the two groups using chi square or Mann–Whitney U testing. Discontinuous (piecewise) growth models [21] allowed estimates of both short-term (birth to NICU discharge) and long-term (post-discharge clinic visits) linear growth trajectories. Separate models assessed change in weight, length, and HC *z*-scores as a function of overall time in months (centered at NICU discharge) and a post-NICU time parameter that had values of zero for all time until NICU discharge, followed by number of months since discharge. The combination of these two parameters afforded the estimation of two growth estimates: (1) Short-term, in-NICU growth (overall time only) and (2) long-term, post-NICU growth (overall time + post-NICU time). The post-NICU time parameter represents the deviation from the in-NICU growth trajectory that occurs after discharge. Models controlled for NICU LOS, GA, sex, group (pre-DM vs. DM), formula proportion, and DM proportion. NICU LOS, GA, sex, and group were mean-centered; formula proportion and DM proportion were not centered. Thus, the overall intercept (*z*-score at NICU discharge) and growth (change in *z*-score over time) parameters represented model estimates for “average” infants (i.e., in terms of NICU LOS, GA, sex, and group) who received 100% MOM (i.e., 0% formula and 0% DM) and thus constituted the reference group. Interaction terms with overall time and the time parameter for all covariates allowed estimates of the effect of the covariates on short-term and long-term growth. To ease interpretation of the model parameters, planned estimates were calculated for short-term and long-term overall trends and for the effects of each covariate on both short-term and long-term growth. All models employed maximum likelihood estimation, as is standard for longitudinal growth models. Under the assumption of missing at random, this allowed unbiased growth trajectories to be estimated for each infant, regardless of missing measurements. For the growth models, we employed the Benjamini–Hochberg approach to control the false discovery rate associated with testing multiple hypotheses [22]. SPSS version 25.0 (IBM SPSS Statistics for Windows, IBM Corporation, Armonk, NY, USA) and SAS version 9.4 (SAS Institute Inc., Cary, NC, USA) were used for the analyses.

## 3. Results

### 3.1. Characteristics of the Sample

Characteristics of the infants and feedings are summarized in Table 1. The demographics of the two groups were similar, as were the frequencies of common NICU morbidities. In the first 14 DOL, infants in the DM group received less MOM, but this difference disappeared for DOL 1–28, with unchanged conclusions when infants fed exclusive (100%) MOM were excluded. The percentage of infants who received exclusive MOM did not differ between the groups. Most formula in the pre-DM group was replaced with DM in the DM group, with only a small percentage of infants receiving formula in the first month of life due to refusal of DM consent. Infants in the DM group achieved a feed volume that allowed discontinuation of parental nutrition earlier than those in the pre-DM group. Retention rates for long-term follow-up were not statistically different between the two groups (n for each time point shown in Table 2). When compared to infants with ≤1 follow-up clinic visit, infants who attended ≥2 of 3 follow-up clinic visits had a longer NICU LOS (85.6 vs. 74.9 days), higher MOM proportion for DOL 1–28 (0.73 vs. 0.62), and were more likely to be female (52.3% vs. 35.6%), *p* < 0.05 for all.

### 3.2. Growth Parameters

Anthropometrics are reported for both groups from birth to 20–24 months CA in Table 2.

**Figure 1:**
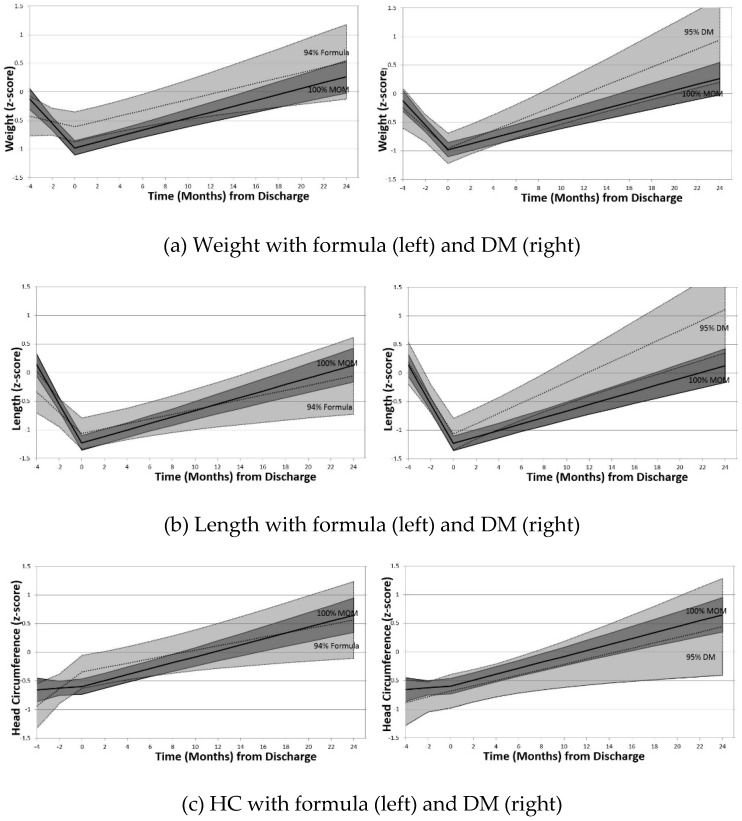
Model estimated anthropometric (*z*-score) changes over time comparing infants at 100% MOM feeds to infants at 95th percentile of cohort for formula or DM feeds. DM: Donor milk; HC: Head circumference. Mean *z*-score: Central solid (MOM) and dotted (formula or DM) lines; 95th% confidence intervals: Shaded areas (dark grey MOM, light grey formula or DM) bordered by solid (MOM) and dotted (formula or DM) lines.

*Z*-scores declined significantly for weight and length during NICU hospitalization from birth to discharge for the cohort as a whole (Table 3). Longer LOS (for weight and length) and higher GA at birth (for all parameters) were associated with a slower rate of decline in short-term growth trajectories. A higher formula proportion was associated with slower rates of decline in short-term growth trajectories for weight and length. DM proportion was not associated with short-term growth for any measure. Figure 1 depicts plots for model-estimated *z*-scores that compare estimates for the reference (100% MOM) infants to estimates for infants at the 95th percentile of formula use for the cohort (which corresponded to receiving 94% formula) or the 95th percentile of DM use (which corresponded to receiving 95% DM).

After NICU discharge, overall growth *z*-scores increased significantly for all parameters (Table 3 and Figure 1). Longer NICU LOS was associated with a slower increase in *z*-score for HC only in initial analysis, but this result was non-significant after correcting for multiple hypothesis testing [22]. No associations between in-NICU supplemental feeding type (formula or DM) and long-term growth were detected (Figure 1).

## 4. Discussion

In this cohort of VLBW infants who received large proportions of MOM, we did not find an association between fortified DM and short-term growth, results which were similar to those from the DoMINO trial, which randomized infants to DM or formula feedings as supplementation to MOM [9]. To our knowledge, this is one of the first contemporary reports of long-term growth in a DM-fed population of VLBW infants in which DM was predominantly a supplement, not a substitute, for MOM. Although Morley et al. conducted a randomized trial comparing long-term growth in premature infants fed unfortified DM vs. preterm formula in the 1980s, these data have limited generalizability because of significant changes in nutritional practices over the past several decades [7,8,15]. Of note, Morley reported no difference in growth at 9 or 18 months post-term, nor at 7.5–8 years of age, but the specific proportions of MOM vs. DM vs. formula feedings were not reported [15]. Madore et al. reported slower weight gain in the first month of life with DM feedings in a small observational cohort of very preterm infants who were fed either exclusive MOM, predominantly formula or predominantly DM for the first month of life. However, continued study of this cohort demonstrated no difference in any growth parameter from 60 days of life to 2 years CA [12]. While the DoMINO trial has recently reported neurodevelopmental follow-up at 18 months in DM-fed infants, long-term growth has not been reported [9].

Previous reports of poor early growth with DM, especially high-dose DM, likely reflect its nutritional inferiority compared to preterm MOM [3]. DM is collected from mothers of term infants who are often several months post-partum, undergoes pasteurization and freeze–thaw cycles, and is fed artificially through feeding tubes that trap nutrients. Thus, DM is typically lower in protein, lipid, sodium, and growth factor concentrations than preterm MOM [4,23,24], as well as enzymes and hormones that play a role in growth, metabolism, and nutrient absorption [25,26,27,28,29]. Given reports of inferior growth with DM [4,7,10,11,12,13] and concerns regarding low protein content [23], our feeding protocol supplemented all DM with protein in addition to standard fortification, regardless of infant growth. Although we did not analyze milk samples, this protein supplementation presumably increased the nutritional content of DM and may explain the lack of effect of DM on short-term growth [30], providing additional evidence to support this widely-used practice for DM. Similar to the DoMINO trial [9], the majority of infant feedings in both groups consisted of MOM, even when excluding infants fed exclusive MOM, thus limiting our ability to study the effects of high-dose DM.

Infants receiving exclusive MOM during the first month of life experienced slower short-term growth when compared to infants who received formula, similar to previous reports of slower growth with increasing proportions of MOM intake [11,31,32]. However, after discharge, exclusive MOM-fed infants in our cohort experienced similar growth trajectories with increasing *z*-scores, thus receiving the short- and long-term benefits from high-dose MOM without significantly affecting later growth [33]. These findings are important given the positive association between growth and neurodevelopment [34] as well as new evidence that early growth in VLBW infants affects long-term metabolic programming and body composition [35,36]. A likely explanation for similar postnatal growth patterns for DM-fed and formula-fed infants is that DM was not used as a long-term diet. Once DM was weaned at about 35 weeks CGA, these infants were fed partial or exclusive formula. Over half of the entire cohort was primarily formula-fed upon NICU discharge (Table S1). Another explanation for the post-discharge change in growth *z*-score is that only a minority (<20%) of infants were still receiving MOM at the 4- and 8-month CA follow-up visits (Table S1), resulting in significant post-discharge “contamination” of the previously defined feeding groups. Future studies are needed to study the growth of VLBW infants who more closely adhere to the World Health Organization’s recommended 6 months of exclusive MOM feeding. However, our cohort is representative of the fact that mothers of preterm infants experience long-term lactation challenges and are much less likely to meet this recommendation [37,38,39].

We were surprised to find a positive association between LOS and short-term growth for weight and length. Brownell et al. also recently reported a positive association between LOS and in-NICU weight *z*-scores [13]. Although we only had detailed in-NICU feeding data for the first 28 DOL, which is a limitation, we speculate that this positive association may be due to decreasing rates of MOM feedings over time, since mothers’ provision of MOM declines with increasing duration of hospitalization [40]. Additionally, while in hospital, infants’ growth was carefully managed by increasing calories as necessary, this intensive practice was impossible after NICU discharge. Although we cannot exclude possible confounding due to other factors that we have not accounted for in our models, such as NICU morbidities, these morbidities were rare (<6% of all subjects) and did not differ between the groups. A study limitation is that bronchopulmonary dysplasia (BPD), a common NICU morbidity that may affect growth, was not included due to incomplete data. The association between LOS and growth outcomes is not simply due to lower birth GA and resultant longer hospitalization, since GA was independently associated with some growth outcomes. Infants with higher GA experienced more normal short-term growth, as shown by a lesser decrease in *z*-scores for all parameters, likely due to a less challenging clinical course and lower nutritional requirements than infants with lower GAs and birth weights [41].

The proportion of feedings equal to MOM in the first 14 DOL was lower in the DM (0.77 MOM) than in the pre-DM group (0.85 MOM), despite a unit policy of a two-day “waiting period” prior to consenting for DM to encourage maternal pumping and MOM use [42]. In NICUs that use DM immediately, the proportion of MOM in the first 14 DOL and incidence of first feed as MOM may be even lower. The risks and benefits of immediate feeds of DM vs. waiting a brief period for MOM colostrum have yet to be determined. However, similar to Marinelli et al. [43], using DM resulted in earlier feeding, more HM and enteral intake in the first 14 days, and fewer days with a central line, with infants reaching solely enteral feeds (no total parenteral nutrition (TPN) or iv fluids) on average 6 days faster (Table 1). This practice likely resulted in lower macronutrient provision very early in life due to fewer days of TPN in the DM group. However, DM group infants received fortified MOM or DM for a longer period than pre-DM infants, reaching full feedings and fortification sooner. It is plausible that these early differences in TPN duration and HM fortification may have balanced each other, since we detected no growth differences by group.

Our study is one of the first to examine the long-term, post-NICU growth implications of fortified DM to supplement an enteral diet with a high proportion of MOM in the critical first month of life. A strength of our study is the racially and ethnically diverse urban cohort of VLBW infants with detailed daily feeding data for the first month of life, as well as multiple growth data points during and after the NICU stay through to 20–24 months CA. However, although we had a relatively large sample size of 321 infants, it was a single center study with a strong MOM-feeding program, so the results may not be as applicable to NICUs with lower MOM and higher DM use in early life [44]. A limitation is loss to follow-up in this high-risk, urban cohort (Table 2), although follow-up rates did not differ between groups. Infants who came to at least 2 follow-up visits differed slightly in characteristics, such as higher in-NICU MOM dose, in keeping with previous follow-up studies at our center. Although these differences are a study limitation, previous studies from our NICU did not find an association between NICU clinic follow-up rates at 20–24 months CA and NICU morbidities that could affect growth [33,45]. In addition, the variables that differed between infants with higher vs. lower follow-up rates (NICU LOS, MOM proportion, sex) were accounted for in the growth model.

Our institutional practice was to discharge infants on unfortified MOM and/or transitional 22 kcal/oz (0.73 kcal/mL) formula, which may differ from other institutions that continue MOM fortification post-discharge. Since published and unpublished studies have not consistently demonstrated beneficial effects of post-discharge fortification on long-term growth in very preterm infants, it is undetermined if our results would have been impacted by different post-discharge fortification practices [46,47,48]. A limitation is that we did not have detailed feeding data other than primary liquid diet type after discharge, but as infants were not subject to any specific nutritional management after discharge, their growth likely reflects a typical infant and toddler diet, so it should be generalizable to a large proportion of NICU graduates.

## 5. Conclusions

VLBW infant weight and length *z*-scores declined during the NICU hospitalization, with no relationship detected as a function of DM proportion, but with a positive association between growth *z*-score and formula proportion. However, after discharge, the overall growth pattern reversed from decreasing to increasing *z*-scores without variation by in-NICU feeding type. These findings support the use of DM early in the post-birth period to mitigate potential risk from formula without compromising long-term growth. Although fortified DM in this cohort did not appear to negatively impact growth through to the age of two years, additional studies are needed to delineate longer-term implications of DM feeding in VLBW infants.

## Figures and Tables

**Table 1:** Cohort characteristics: Median (IQR) or *n* (%).

		Pre-DM *n* = 160	DM *n* = 161	*p* value pre-DM vs. DM Groups *
Birth weight (g)		1050 (750, 1220)	1000 (800, 1180)	0.80
Gestational age (weeks)		27.7 (26.1, 29.4)	27.7 (25.9, 29.3)	0.73
Female sex		72 (45.0)	74 (46.0)	0.86
Small for gestational age		27 (16.9)	21 (13.0)	0.34
Race/ethnicity	White	33 (20.6)	34 (21.1)	0.97
	Black	89 (55.6)	88 (54.7)	
	Hispanic	34 (21.3)	36 (22.4)	
	Other	4 (2.5)	3 (1.9)	
NICU length of stay (days)		71.0 (47.5, 106.0)	77.0 (52.5, 104.8)	0.43
Exclusive MOM feeds DOL 1–28		33 (20.6)	33 (20.5)	0.98
First feed MOM		144 (90.0)	116 (72.5)	**<0.01**
Entire cohort (*n* = 321)	Proportion MOM DOL 1–14	1.00 (0.92, 1.00)	1.00 (0.59, 1.00)	**0.01**
	Proportion MOM DOL 1–28	0.97 (0.35, 1.00)	0.99 (0.35, 1.00)	0.83
	Proportion DM DOL 1–14	0 (0, 0)	0 (0,0.40)	**<0.01**
	Proportion DM DOL 1–28	0 (0, 0)	0.01 (0, 0.63)	**<0.01**
	Proportion formula DOL 1–14	0 (0, 0.08)	0 (0, 0)	**<0.01**
	Proportion formula DOL 1–28	0.03 (0, 0.65)	0 (0, 0)	**<0.01**
Cohort excluding infants fed exclusive MOM in first 28 DOL (*n* = 255)	Proportion MOM DOL 1–14	1.00 (0.85, 1.00)	0.97 (0.46, 1.00)	**<0.01**
	Proportion MOM DOL 1–28	0.84 (0.26, 1.00)	0.68 (0.11, 1.00)	0.48
	Proportion DM DOL 1–14	0 (0, 0)	0.02 (0, 0.51)	**<0.01**
	Proportion DM DOL 1-28	0 (0, 0)	0.32 (0, 0.79)	**<0.01**
	Proportion formula DOL 1-14	0 (0, 0.15)	0 (0, 0)	**<0.01**
	Proportion formula DOL 1-28	0.16 (0, 0.75)	0 (0, 0)	**<0.01**
First DOL of solely enteral feeds (no TPN/IVFs)		20.0 (15.0, 29.5) (*n* = 157)	14.0 (12.0, 23.0) (*n* = 147)	**<0.01**
Severe ROP (≥ stage 3)		0 (0)	0 (0)	1
Significant brain Injury: severe IVH (≥ grade III) or PVL		7 (4.4)	9/158 (5.7)	0.59
NEC (≥ stage 2)		9 (5.6)	3/160 (1.9)	0.08
Attended ≥ 2 of 3 NICU follow-up clinic visits		106 (66.3)	91/155 (58.7)	0.17

MOM: Mother’s own milk; DOL: Day of life; DM: Donor milk; TPN: Total parenteral nutrition; IVFs: Intravenous fluids; ROP: Retinopathy of prematurity; IVH: Intraventricular hemorrhage; PVL: Periventricular leukomalacia; NEC: Necrotizing enterocolitis. “Proportion:” Calculated for each infant as the cumulative volume of MOM, DM, or formula received divided by total volume of enteral feeds (MOM + DM + formula) received over the specified time period. * Chi square or Mann–Whitney U test, bold *p* values: significant at *p* ≤ 0.0.5

**Table 2:** Longitudinal anthropometrics for cohorts.

		Pre-DM	DM
Birth *n* = 321 (160 pre-DM; 161 DM)	Weight (g)	997 ± 245	998 ± 278
	Length (cm)	35.3 ± 3.1	35.5 ± 3.7
	HC (cm)	24.8 ± 2.1	24.9 ± 2.5
32 weeks corrected GA *n* = 307 (154 pre-DM; 153 DM)	Weight (g)	1442 ± 254	1380 ± 197
	Length (cm)	39.5 ± 2.0	39.6 ± 2.3
	HC (cm)	27.5 ± 1.4	27.4 ± 1.3
NICU Discharge *n* = 315 (160 pre-DM; 155 DM)	Weight (g)	2946 ± 901	2834 ± 981
	Length (cm)	47.1 ± 4.1	47.2 ± 4.4
	HC (cm)	33.4 ± 2.6	33.2 ± 2.6
4 months CA *n* = 215 (116 pre-DM; 99 DM)	Weight (g)	6477 ± 1022	6303 ± 1022
	Length (cm)	61.5 ± 3.0	61.3 ± 3.5
	HC (cm)	41.6 ± 1.7	41.0 ± 2.0
8 months CA *n* = 200 (108 pre-DM; 92 DM)	Weight (g)	8133 ± 1160	7840 ± 1196
	Length (cm)	68.9 ± 3.1	68.2 ± 3.4
	HC (cm)	44.3 ± 1.7	43.7 ± 1.9
20–24 months CA *n* = 156 (91 pre-DM; 65 DM)	Weight (g)	11,168 ± 1797	11,017 ± 1476
	Length (cm)	82.7 ± 4.2	83.1 ± 3.4
	HC (cm)	47.4 ± 1.8	46.9 ± 1.7

DM: Donor milk; HC: Head circumference; GA: Gestational age; CA: Corrected age.

**Table 3:** Short- and long-term changes in growth parameters.

	Weight (*z*-score)		Length (*z*-score)		Head Circumference (*z*-score)	
	Parameter Estimate (SE)	*p* ^3^	Parameter Estimate (SE)	*p* ^3^	Parameter Estimate (SE)	*p* ^3^
**Short-term growth (in-NICU: birth to discharge)**						
Growth for reference infant ^1^	−0.216 (0.029)	**<0.001**	−0.333 (0.027)	**<0.001**	0.013 (0.030)	0.654
Deviation from reference growth						
NICU LOS ^2^	0.017 (0.004)	**<0.001**	0.018 (0.003)	**<0.001**	0.006 (0.003)	0.090
Birth gestational age ^2^	0.043 (0.013)	**0.001**	0.035 (0.012)	**0.003**	0.047 (0.013)	**<0.001**
Female sex ^2^	0.000 (0.040)	0.992	0.066 (0.035)	0.060	−0.051 (0.038)	0.181
Proportion formula ^2^	0.018 (0.007)	**0.009**	0.016 (0.006)	**0.010**	0.013 (0.007)	0.057
Proportion DM ^2^	0.004 (0.007)	0.562	0.004 (0.006)	0.467	0.003 (0.006)	0.591
**Long-term growth (post-NICU: discharge to 20–24 months corrected age)**						
Growth for reference infant ^1^	0.052 (0.006)	**<0.001**	0.057 (0.006)	**<0.001**	0.052 (0.006)	**<0.001**
Deviation from reference growth						
NICU LOS ^2^	−0.002 (0.001)	0.082	−0.001 (0.001)	0.397	−0.003 (0.001)	0.037 ^4^
Birth gestational age ^2^	0.001 (0.003)	0.788	0.002 (0.004)	0.654	−0.002 (0.004)	0.555
Female sex ^2^	−0.002 (0.009)	0.840	−0.005 (0.010)	0.630	−0.002 (0.010)	0.864
Proportion formula ^2^	0.000 (0.002)	0.762	−0.002 (0.002)	0.347	−0.001 (0.002)	0.392
Proportion DM ^2^	0.003 (0.002)	0.075	0.003 (0.002)	0.064	−0.001 (0.002)	0.693

DM: Donor milk; LOS: Length of stay; NICU: Neonatal intensive care unit. Time measured in months, centered at NICU discharge date. ^1^ Average monthly change in z-score for reference subjects who received 100% mother’s own milk (no formula or DM) at mean NICU length of stay (NICU LOS), gestational age, and sex. ^2^ Deviation from the reference growth trend for each week (NICU LOS, GA), female sex, and for each 10% increase in proportion of formula or DM, respectively. ^3^ *p*-value testing hypothesis that the change in z-score is zero, bold p values: significant at *p* ≤ 0.05. ^4^ Nonsignificant at *p* < 0.05 after correcting for multiple hypothesis testing using procedures specified by Benjamini and Hochberg [22].

## Supplementary Materials

The following are available online at http://www.mdpi.com/2072-6643/11/2/241/s1, Table S1: Primary liquid diet at and after NICU discharge.
