# Supplementary material for: Impact of Donor Milk on Short- and Long-Term Growth of Very Low Birth Weight Infants
Source: Nutrients. 2019 Jan 22;11(2):241. doi: 10.3390/nu11020241 (PMC6412258; doi:10.3390/nu11020241)
Supplement: Supplementary file 1 [file nutrients-11-00241-s001.pdf]

Table S1: Primary liquid diet at and after NICU discharge

	MOM (%)	Formula (%)	Whole Cow's Milk (%)
**NICU discharge**	37	63	0
**4 months CA**	12	88	0
**8 months CA**	7	83	9
**20-24 months CA**	0	12	88

MOM: mother's own milk; CA: corrected age
